# Supplementary material for: Evidence supported by Mendelian randomization: impact on inflammatory factors in knee osteoarthritis
Source: Front Med (Lausanne). 2024 May 28;11:1382836. doi: 10.3389/fmed.2024.1382836 (PMC11165061; doi:10.3389/fmed.2024.1382836)
Supplement: Supplementary file 1 [file Data_Sheet_1.ZIP › data/GCST90274832_5e6/832.leaveoneout.pdf]

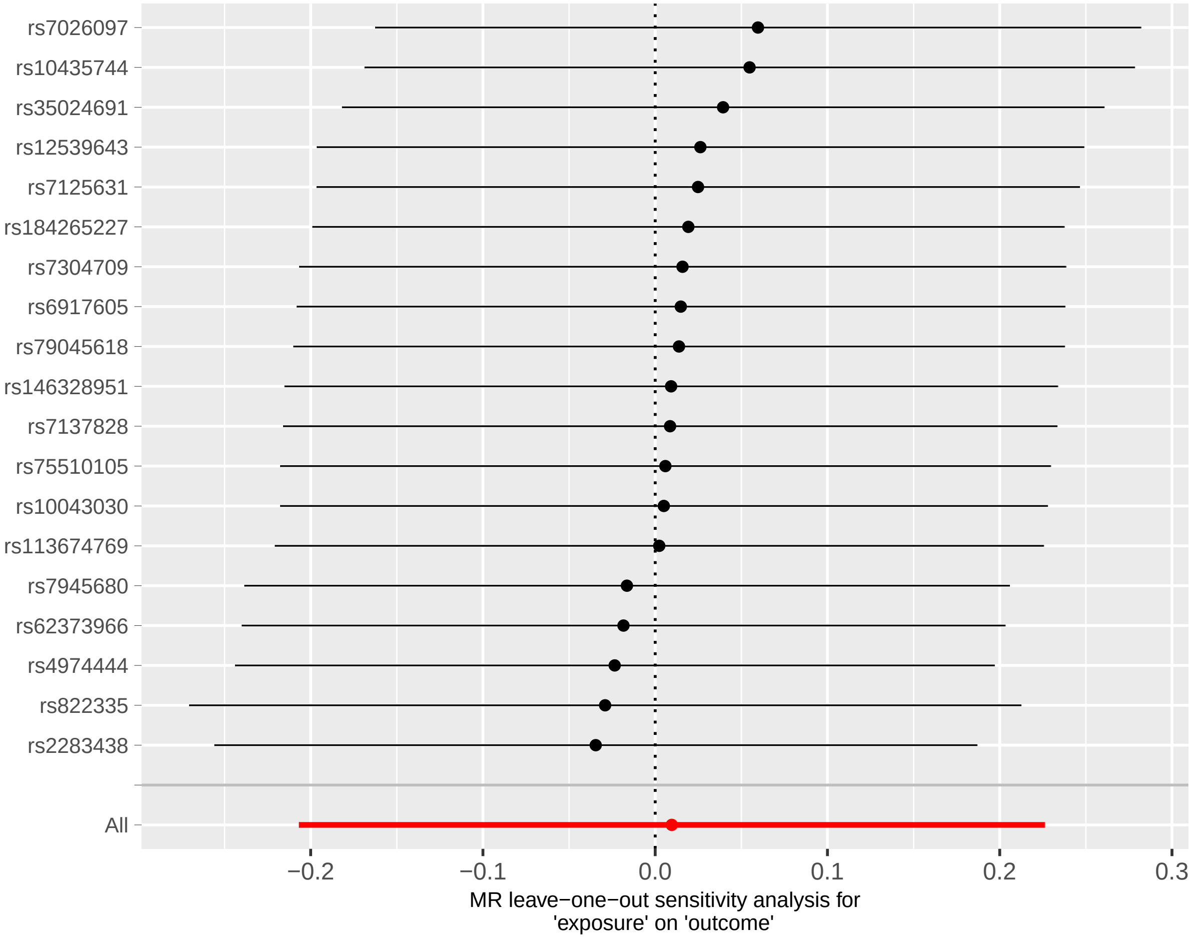
rs7026097
rs10435744
rs35024691
rs12539643
rs7125631
rs184265227
rs7304709
rs6917605
rs79045618
rs146328951
rs7137828
rs75510105
rs10043030
rs113674769
rs7945680
rs62373966
rs4974444
rs822335
rs2283438
All
−0.2
−0.1
0.0
0.1
0.2
0.3
MR leave−one−out sensitivity analysis for
'exposure' on 'outcome'
